# Supplementary material for: Cell surface patching via CXCR4-targeted nanothreads for cancer metastasis inhibition
Source: Nat Commun. 2024 Mar 29;15:2763. doi: 10.1038/s41467-024-47111-z (PMC10980815; doi:10.1038/s41467-024-47111-z)
Supplement: Supplementary file 1 — Supplementary Information [file 41467_2024_47111_MOESM1_ESM.pdf]

Supplementary Information for

Cell Surface patching via CXCR4-targeted Nanothreads for Cancer Metastasis Inhibition

Minglu Zhou[1, #], Chendong Liu[1, #], Bo Li[1], Junlin Li[1], Ping Zhang[1], Yuan Huang[1], Lian Li[1 ✉]

[1]Key Laboratory of Drug-Targeting and Drug Delivery System of the Education Ministry and Sichuan Province, Sichuan Engineering Laboratory for Plant-Sourced Drug and Sichuan Research Center for Drug Precision Industrial Technology, West China School of Pharmacy, Sichuan University, Chengdu 610041, China.

[#]These authors contributed equally: Minglu Zhou, Chendong Liu.

Correspondence:

Lian Li, E-mail: liliantripple@163.com

This file includes:

Supplementary Fig. 1 Synthesis routes for nanothreads.

Supplementary Fig. 2 Representative confocal microscopy image of non-targeted P-CM1-Cy5 treated 4T1 cells.

Supplementary Fig. 3 Specificity of BS to CXCR4.

Supplementary Fig. 4 Representative confocal microscopy image of EGFP-CXCR4-transfected 4T1 cells.

Supplementary Fig. 5 Calcium influx in 4T1 cells during CXCL12 stimulation.

Supplementary Fig. 6 In vitro anti-metastasis analysis.

Supplementary Fig. 7 Biodistribution analysis of Nanothread-1 and Nanothread-2.

Supplementary Fig. 8 Serum chemistry and hematological cell studies of healthy mice received treatment with P-BS-CM1→P-CM2.

Supplementary Fig. 9 Representative hematoxylin and eosin staining of major organs of healthy mice received treatment with P-BS-CM1→P-CM2.

Supplementary Fig. 10 Tumor accumulation and pharmacokinetics evaluation of Nanothread-1 and Nanothread-2 upon consecutive delivery, simultaneous delivery, and post-assembly delivery.

Supplementary Fig. 11 Pharmacokinetic analysis of Nanothread-1 and Nanothread-2 at each cycle in a four-round treatment regimen.

Supplementary Fig. 12 Representative histological images of fiber collagens.

Supplementary Fig. 13 Immunofluorescence staining and semi-quantitative analysis of MMP-9 and TGF-β in tumor tissues.

Supplementary Fig. 14 Immunofluorescence staining and semi-quantitative analysis of LOX in lung tissues.

Supplementary Fig. 15 Immunofluorescence staining and semi-quantitative analysis of E-cadherin in lung tissues.

Supplementary Fig. 16 Flow cytometry gating strategies and analyses for $CD8^+$ T lymphocytes, Tregs and MDSCs in tumor tissues.

Supplementary Fig. 17 Analysis of PI3K pathway in 4T1 cells.

Supplementary Fig. 18 Individual tumor growth without laser irradiation.

Supplementary Fig. 19 Individual tumor growth with laser irradiation.

Supplementary Figure 20. Correlation between pulmonary BMDCs and CXCL12.

Supplementary Figure 21. Flow cytometry gating strategies and analyses for $CD8^+$ T lymphocytes, Tregs and MDSCs in tumor tissues with laser irradiation.

Supplementary Figure 22. Individual tumor growth upon $CD8^+$ T cell ablation.

Supplementary Table 1. Characterization of various synthetic copolymers.

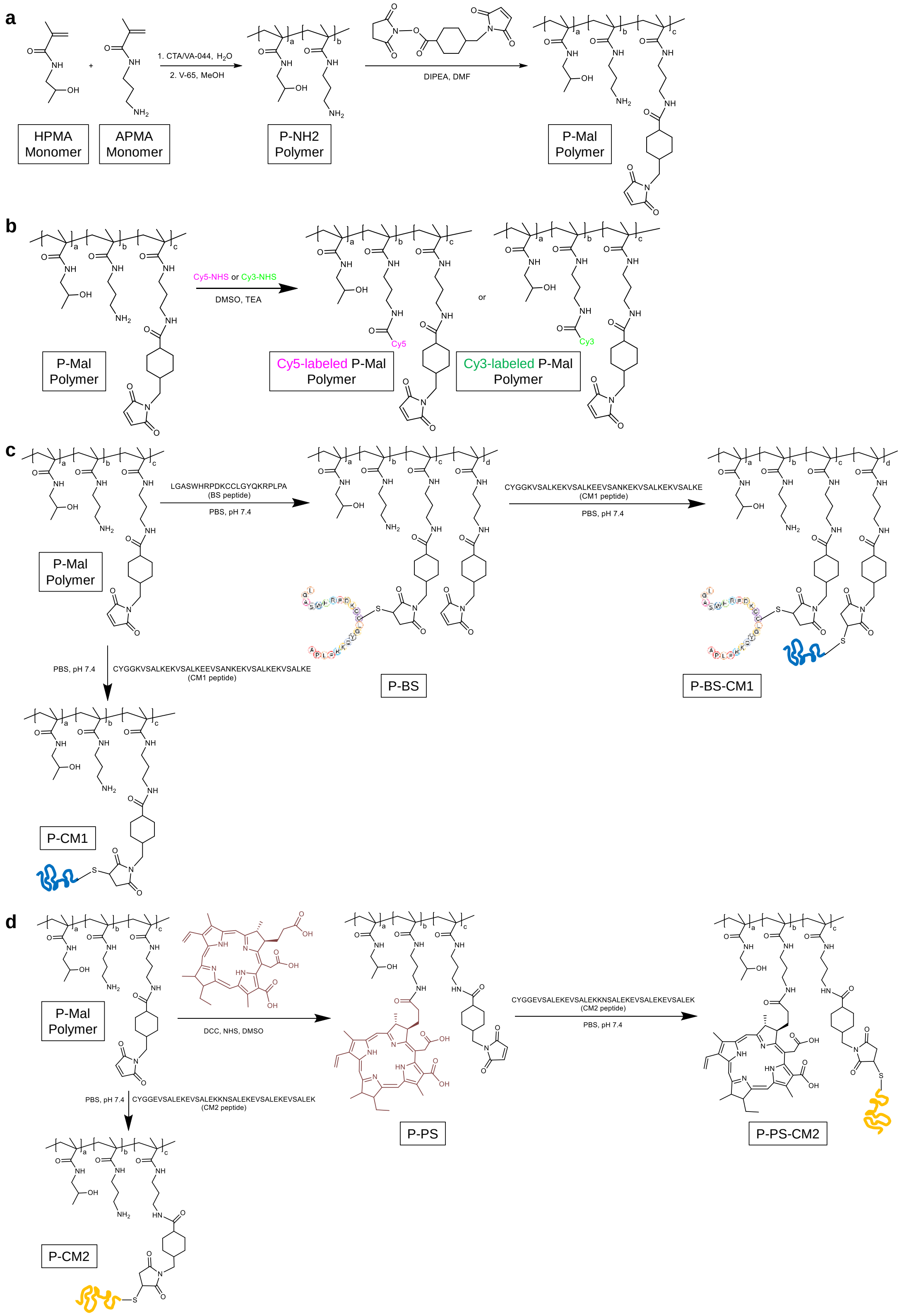

Supplementary Fig. 1 | Synthesis routes for **a,** polymer precursor P-Mal, **b,** fluorescence-labeled P-Mal, **c,** Nanothread-1 (P-BS-CM1), and **d,** Nanothread-2 (P-CM2 or P-PS-CM2). Created with ChemDraw Professional 16.0 software.

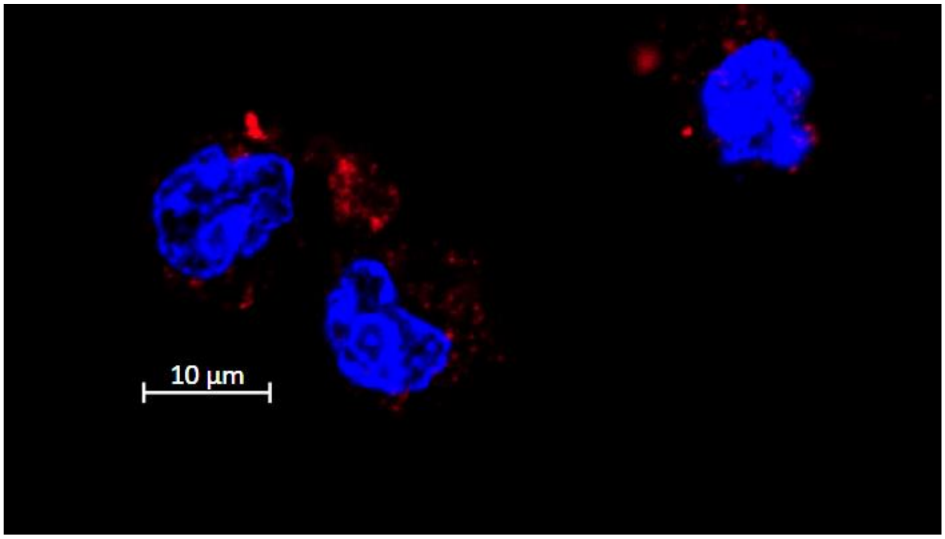

Supplementary Fig. 2 | Representative confocal microscopy image of non-targeted P-CM1-Cy5 treated 4T1 cells. 4T1 cells were treated with P-CM1-Cy5 for 1 h, and then incubated in fresh culture medium for 1 h. Blue indicates cell nuclei, red indicates Cy5. Scale bars, 10 μm. The experiment was repeated three times independently with similar results.

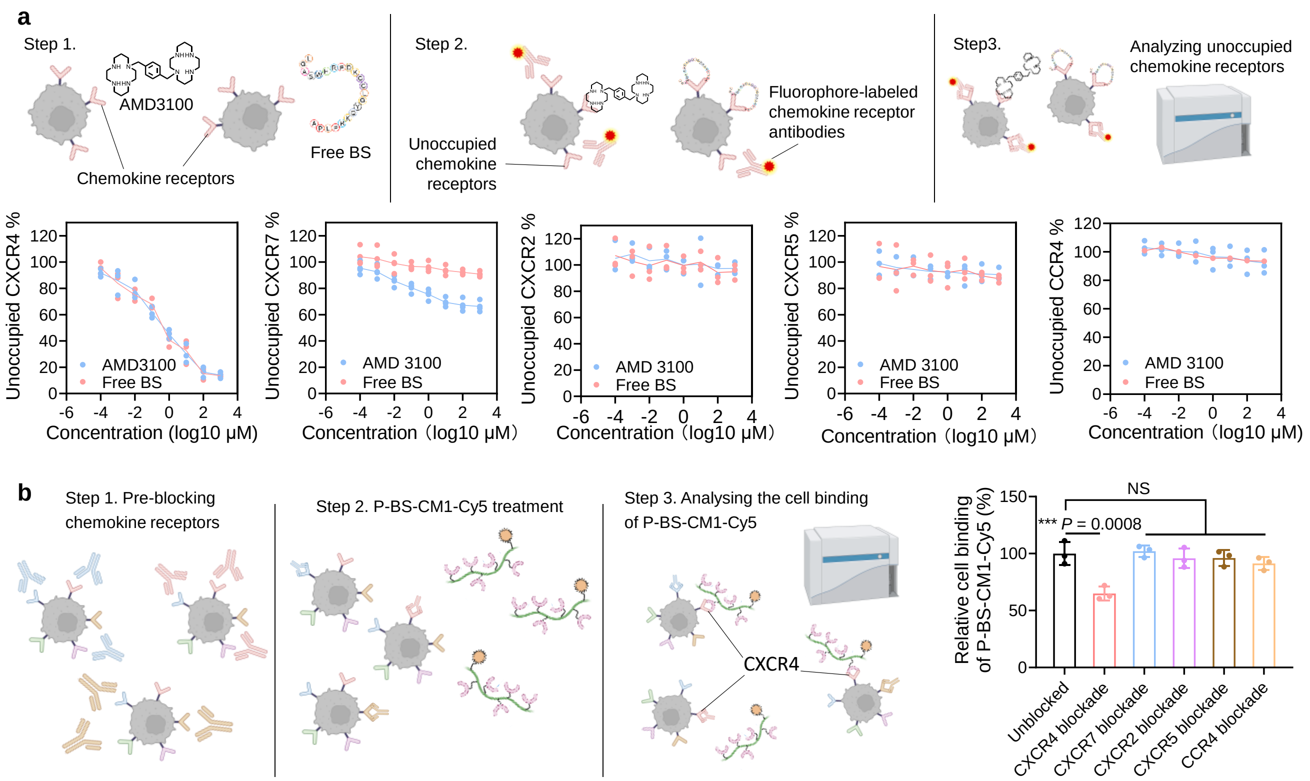

Supplementary Fig. 3 | Specificity of BS to C-X-C motif chemokine receptor 4 (CXCR4). **a,** Dose-dependent chemokine receptor occupation assay. 4T1 cells were exposed to AMD3100 or free BS with concentrations ranging from 0.1 nM to 1 mM equivalent at 25 °C for 1 h to occupy chemokine receptors. Unoccupied chemokine receptors (CXCR4, CXCR7, CXCR2, CXCR5, and CCR4) on cell surface were stained with corresponding PE-labeled antibodies (1:100 dilution, 4 °C, 1 h), prior to flow cytometry analysis. **b,** Chemokine receptors competitive binding assay. 4T1 cells were pre-blocked with antibodies against CXCR4, CXCR7, CXCR2, CXCR5, and chemokine C-C-motif receptor 4 (CCR4), respectively (1:100 dilution, 4 °C, 1 h), and then incubated with P-BS-CM1-Cy5 at 25°C for 1 h. The cell binding of P-BS-CM1-Cy5 was analyzed using flow cytometry. n=3 biologically independent samples per group. Chemical structure of AMD3100 was created with ChemDraw Professional 16.0 software. The experiments were repeated twice independently with similar results. Data are presented as mean ± SD. Statistics are calculated by one-way ANOVA with Tukey’s multiple comparisons test. ***$P < 0.001$. Source data are provided as a Source Data file.

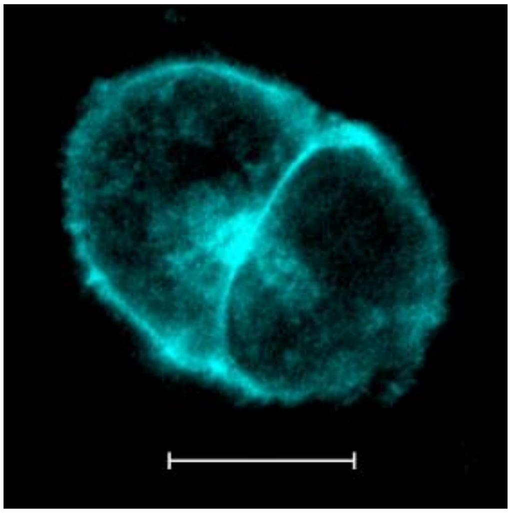

Supplementary Fig. 4 | Representative confocal microscopy image of EGFP-CXCR4-transfected 4T1 cells. CXCR4 fluorescence are enriched at the contact interface between two cells. Cyan indicates EGFP. Scale bar, 20 μm. The experiment was repeated three times independently with similar results.

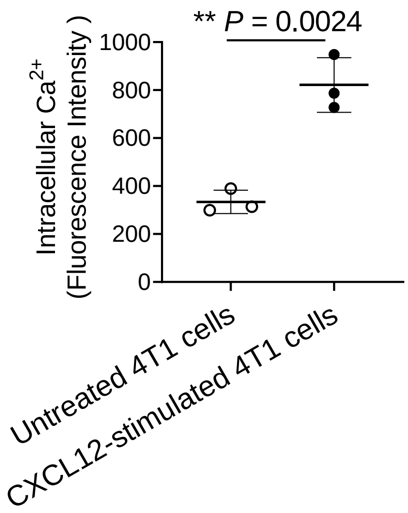

Supplementary Fig. 5 | Calcium influx in 4T1 cells during CXCL12 stimulation. n=3 biologically independent samples per group. The experiment was repeated twice independently with similar results. Data are presented as mean ± SD. Statistics are calculated by unpaired two-tailed Student's t-test. ** $P < 0.01$. Source data are provided as a Source Data file.

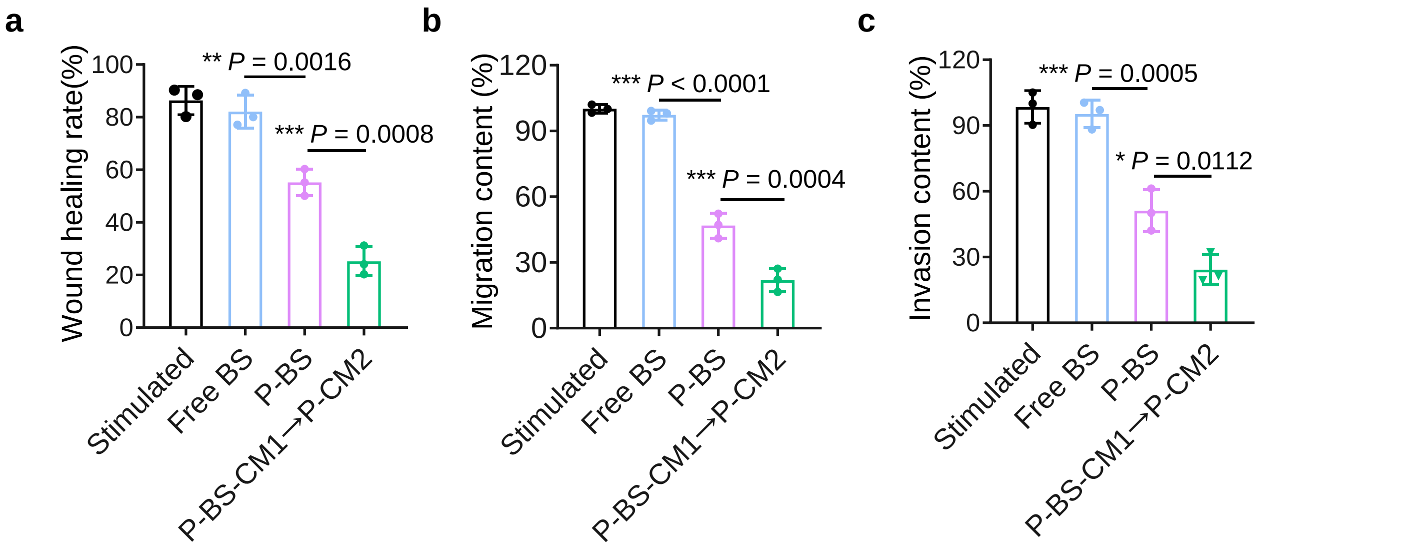

Supplementary Fig. 6 | In vitro anti-metastasis analysis. Quantitative analysis of lateral mobility, longitudinal mobility, and invasiveness of 4T1 cells by **a,** wound healing, **b,** migration, and **c,** invasion assays. n=3 biologically independent samples per group. The experiments were repeated twice independently with similar results. Data are presented as mean ± SD. Statistics are calculated by one-way ANOVA with Tukey's multiple comparisons test. * $P < 0.05$, ** $P < 0.01$, *** $P < 0.001$, **** $P < 0.0001$. Source data are provided as a Source Data file.

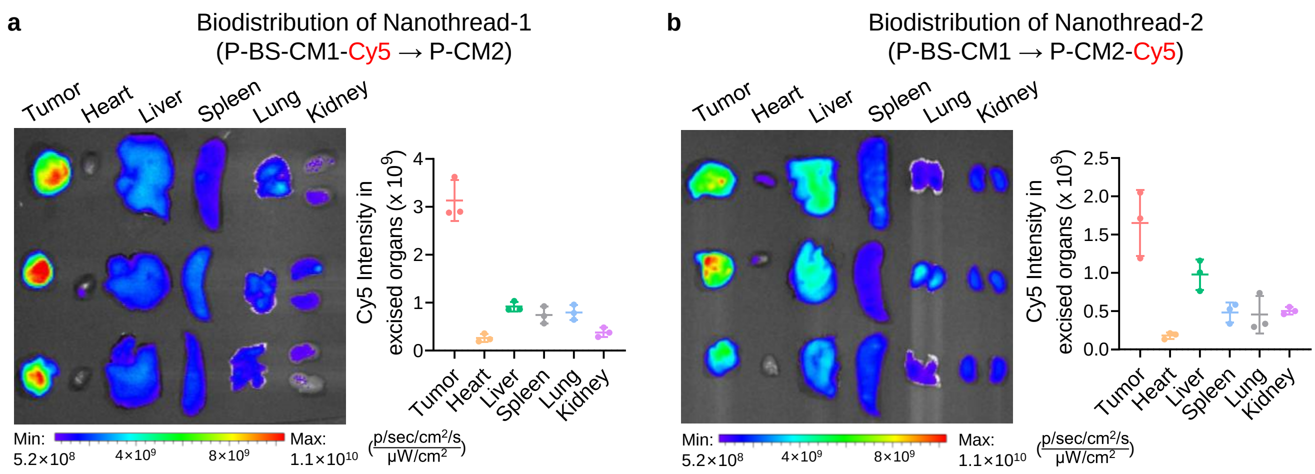

Supplementary Fig. 7 | Biodistribution analysis of Nanothread-1 and Nanothread-2. Orthotopic 4T1 breast tumor bearing female mice were intravenously treated with **a,** P-BS-CM1-Cy5→P-CM2 (24 h time lag), or **b,** P-BS-CM1→P-CM2-Cy5 (24 h time lag). After 72 h post-injection of Cy5-labeled Nanothread-1 or Nanothread-2, major organs were excised for imaging and semi-quantification. n=3 animals per group. Data are presented as mean ± SD. Source data are provided as a Source Data file.

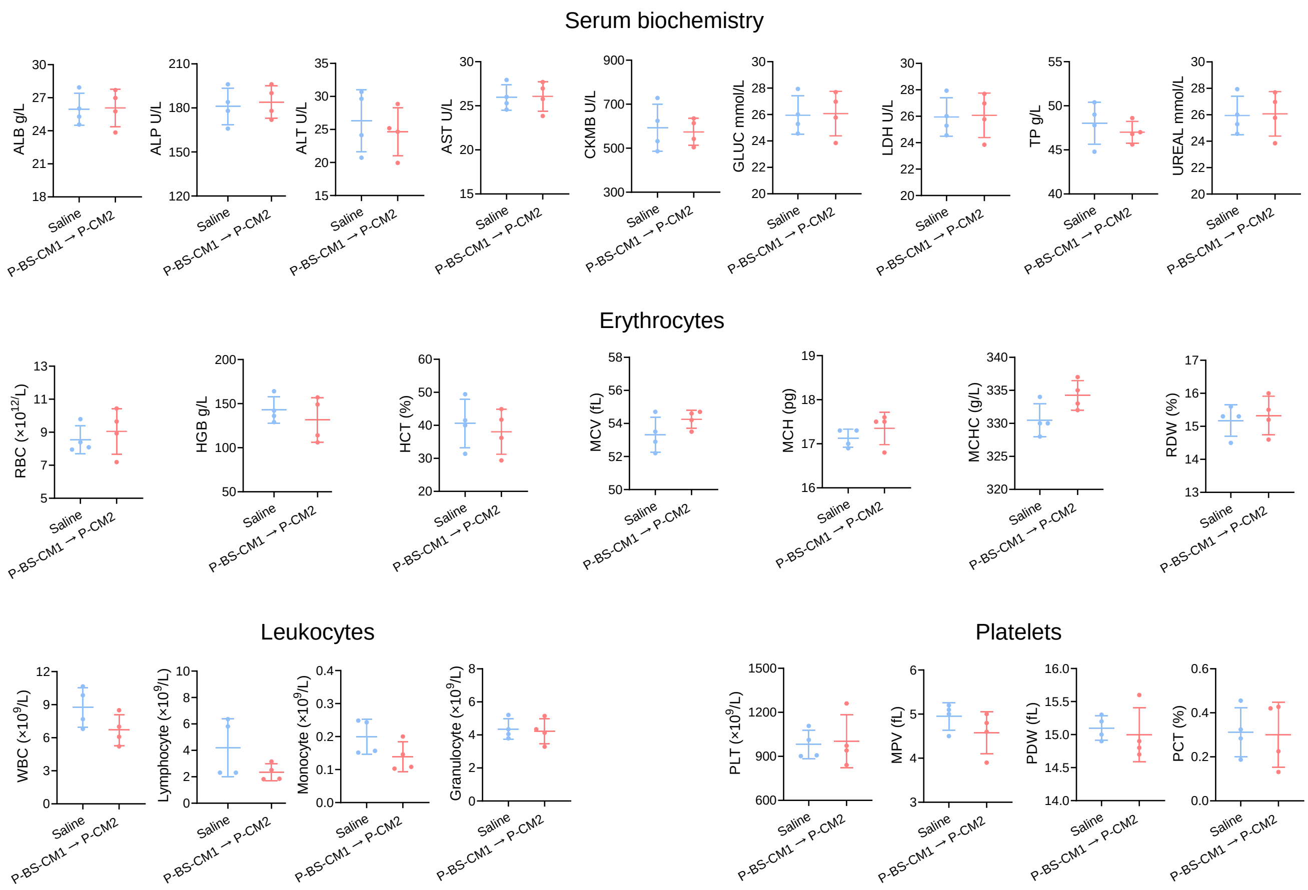

Supplementary Fig. 8 | Serum chemistry, and hematological cell studies at the endpoint after tumor-free female healthy mice received four-cycled weekly treatment with P-BS-CM1→P-CM2. n=4 animals per group. ALB, albumin; ALP, alkaline phosphatase; ALT, alanine aminotransferase; AST, aspartate aminotransferase; CKMB, creatine kinase-MB; GLUC, glucose; LDH, lactate dehydrogenase; TP, total protein. RBC, red blood cell; HGB, hemoglobin; HCT, hematocrit value; MCV, mean corpuscular volume; MCH, mean corpuscular hemoglobin; MCHC, mean corpuscular hemoglobin concentration; RDW, red blood cell volume distribution width; WBC, white blood cell; PLT, platelet; MPV, mean platelet volume; PDW, platelet volume distribution width; PCT, plateletcrit. Data are presented as mean ± SD. Source data are provided as a Source Data file.

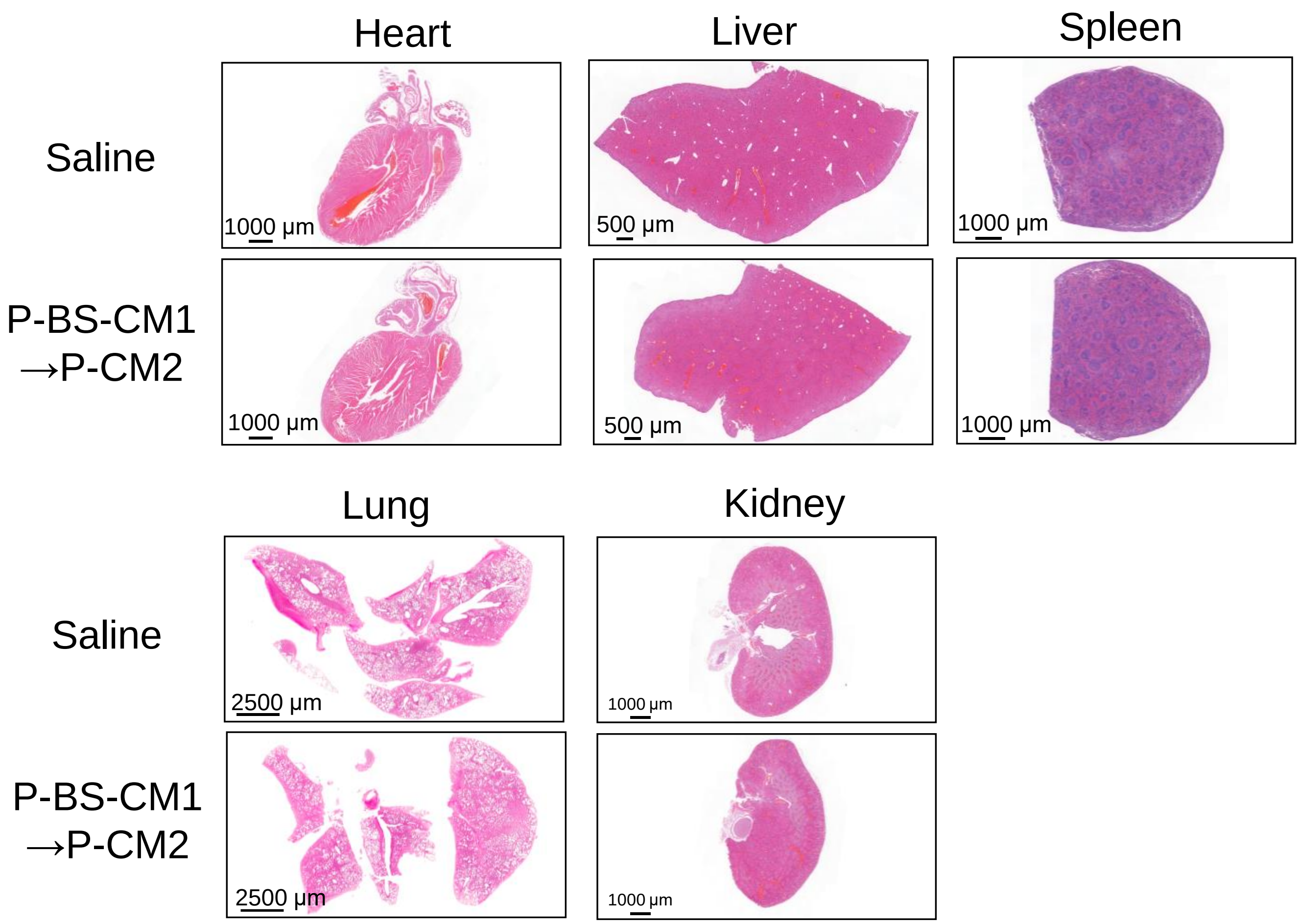

Supplementary Fig. 9 | Representative hematoxylin and eosin staining of major organs (heart, liver, spleen, lung, kidney) at the endpoint after tumor-free female healthy mice received four-cycled weekly treatment with P-BS-CM1→P-CM2. Images are representative of four mice per group.

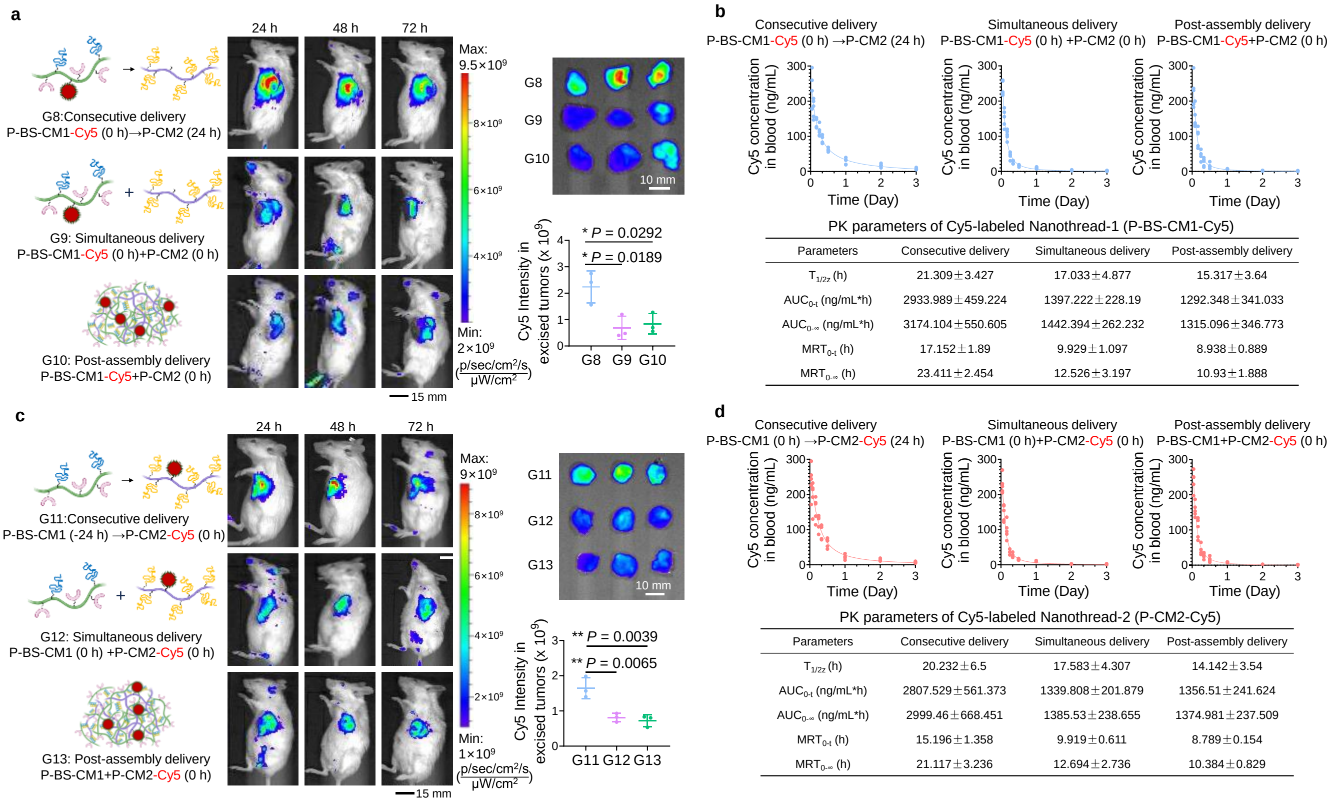

PK parameters of Cy5-labeled Nanothread-1 (P-BS-CM1-Cy5)

Parameters	Consecutive delivery	Simultaneous delivery	Post-assembly delivery
$T_{1/2z}$ (h)	21.309±3.427	17.033±4.877	15.317±3.64
AUC_{0-t} (ng/mL*h)	2933.989±459.224	1397.222±228.19	1292.348±341.033
$AUC_{0-\infty}$ (ng/mL*h)	3174.104±550.605	1442.394±262.232	1315.096±346.773
MRT_{0-t} (h)	17.152±1.89	9.929±1.097	8.938±0.889
$MRT_{0-\infty}$ (h)	23.411±2.454	12.526±3.197	10.93±1.888

PK parameters of Cy5-labeled Nanothread-2 (P-CM2-Cy5)

Parameters	Consecutive delivery	Simultaneous delivery	Post-assembly delivery
$T_{1/2z}$ (h)	20.232±6.5	17.583±4.307	14.142±3.54
AUC_{0-t} (ng/mL*h)	2807.529±561.373	1339.808±201.879	1356.51±241.624
$AUC_{0-\infty}$ (ng/mL*h)	2999.46±668.451	1385.53±238.655	1374.981±237.509
MRT_{0-t} (h)	15.196±1.358	9.919±0.611	8.789±0.154
$MRT_{0-\infty}$ (h)	21.117±3.236	12.694±2.736	10.384±0.829

Supplementary Fig. 10 | Tumor accumulation and pharmacokinetics studies. Representative fluorescence images exhibiting tumor accumulation (**a, c**) and pharmacokinetics evaluation (**b, d**) of Nanothread-1 (**a, b**) and Nanothread-2 (**c, d**) upon consecutive delivery, simultaneous delivery, and post-assembly delivery in orthotopic breast cancer female mouse models. Whole body real-time fluorescence imaging was performed over 72 h post-injection utilizing an IVIS optical imaging system. Fluorescent images of excised tumors were captured at the endpoint and semi-quantified (**a, c**). n=3 animals per group (**a, c**). Pharmacokinetic (PK) parameters of statistical moment analysis are calculated using DAS 2.0 software (**b, d**). n=5 animals per group (**b, d**). $T1/2_Z$: half life, AUC: area under curve, MRT: mean residence time. Data are presented as mean ± SD. Statistics are calculated by one-way ANOVA with Tukey's multiple comparisons test. *$P < 0.05$, **$P < 0.01$. Fluorescent images are representative of three mice per group. Source data are provided as a Source Data file.

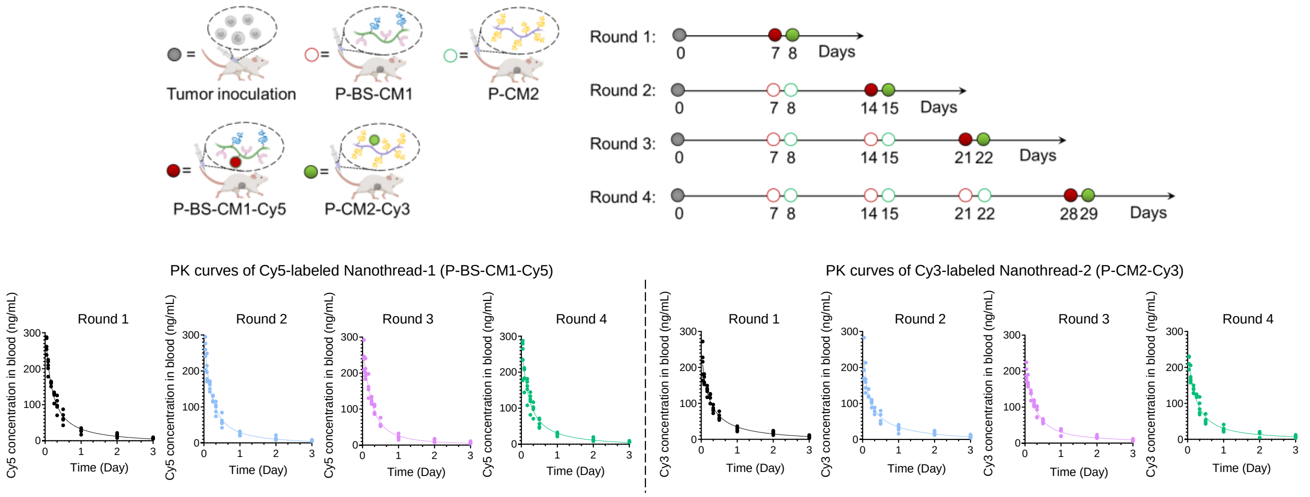

PK parameters of Cy5-labeled Nanothread-1 (P-BS-CM1-Cy5)

Parameters	Round 1	Round 2	Round 3	Round 4
$T_{1/2z}$ (h)	20.103±7.915	19.897±4.552	20.426±8.439	19.385±4.805
AUC_{0-t} (ng/mL*h)	3031.336±530.152	2855.252±543.828	2770.463±509.583	2858.978±506.747
$AUC_{0-\infty}$ (ng/mL*h)	3231.178±614.474	3012.83±586.28	2953.481±678.504	3033.455±583.835
MRT_{0-t} (h)	15.239±1.348	14.589±1.195	14.222±1.603	15.401±1.631
$MRT_{0-\infty}$ (h)	21.021±4.614	19.827±1.137	20.482±4.908	21±3.141

PK parameters of Cy3-labeled Nanothread-2 (P-CM2-Cy3)

Parameters	Round 1	Round 2	Round 3	Round 4
$T_{1/2z}$ (h)	19.104±3.483	18.965±3.022	19.193±3.042	18.712±5.773
AUC_{0-t} (ng/mL*h)	2910.449±479.173	2845.842±670.957	2847.475±463.394	2805.642±501.797
$AUC_{0-\infty}$ (ng/mL*h)	3143.497±607.036	3070.777±804.281	3060.689±565.511	3022.978±666.243
MRT_{0-t} (h)	17.874±1.409	17.76±1.068	17.779±1.27	16.883±2.739
$MRT_{0-\infty}$ (h)	23.669±4.167	23.374±3.332	23.927±3.854	22.801±5.68

Supplementary Fig. 11 | Pharmacokinetic (PK) analysis of Nanothread-1 and Nanothread-2 at each cycle in a four-round treatment regimen post injection of Cy5 labeled Nanothread-1 or Cy3 labeled Nanothread-2. The arrows indicated the treatment regimen. PK parameters of statistical moment analysis are calculated using DAS 2.0 software. n=5 animals per group. $T_{1/2Z}$: half life, AUC: area under curve, MRT: mean residence time. Data are presented as mean ± SD. Source data are provided as a Source Data file.

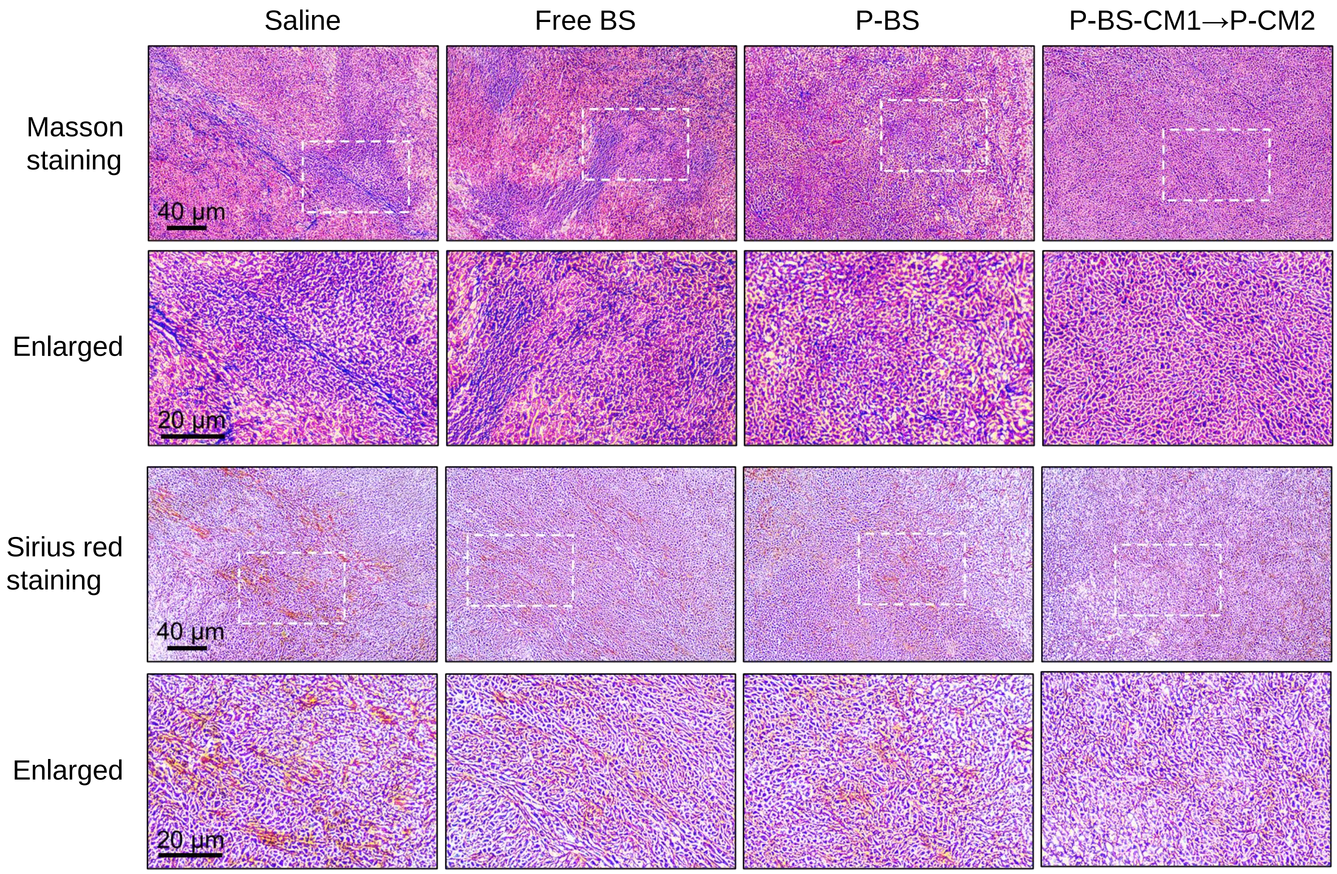

Supplementary Fig. 12 | Representative histological images of fiber collagens using Masson staining and Sirius staining. Spontaneous lung metastasis mouse models of female BALB/c mice bearing orthotopic 4T1 tumors received three cycles of free BS, P-BS, or sequential P-BS-CM1→P-CM2 (24 h lag) on days 7, 14, and 21. Analyses occurred on day 28. White rectangles (top) indicates the enlarged regions (bottom). Images are representative of five mice per group.

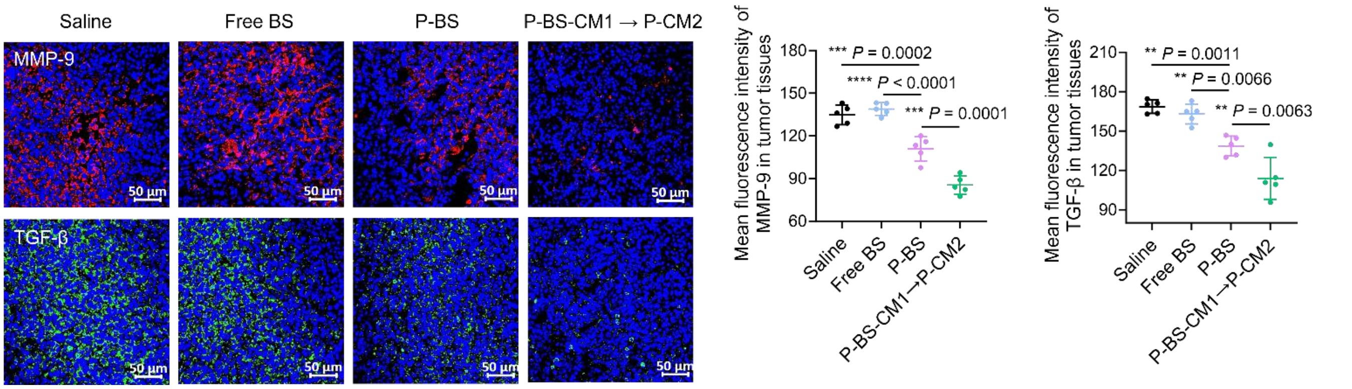

Supplementary Fig. 13 | Immunofluorescence staining and semi-quantitative analysis of typical EMT-promoting factors (MMP-9 and TGF-β) of CXCR4 downstream in tumor tissues of mice on day 28, after treatments with three cycles of free BS, P-BS, and P-BS-CM1→P-CM2 (24 h time lag) on day 7, 14, and 21. Blue, cell nuclei; red, matrix metalloproteinase-9 (MMP-9); green, transforming growth factor-β (TGF-β). Scale bars, 50 μm. Images are representative of five mice per group and analyzed by the Image J software. Data are presented as mean ± SD. Statistics are calculated by one-way ANOVA with Tukey's multiple comparisons test. ** $P < 0.01$, *** $P < 0.001$, **** $P < 0.0001$. Source data are provided as a Source Data file.

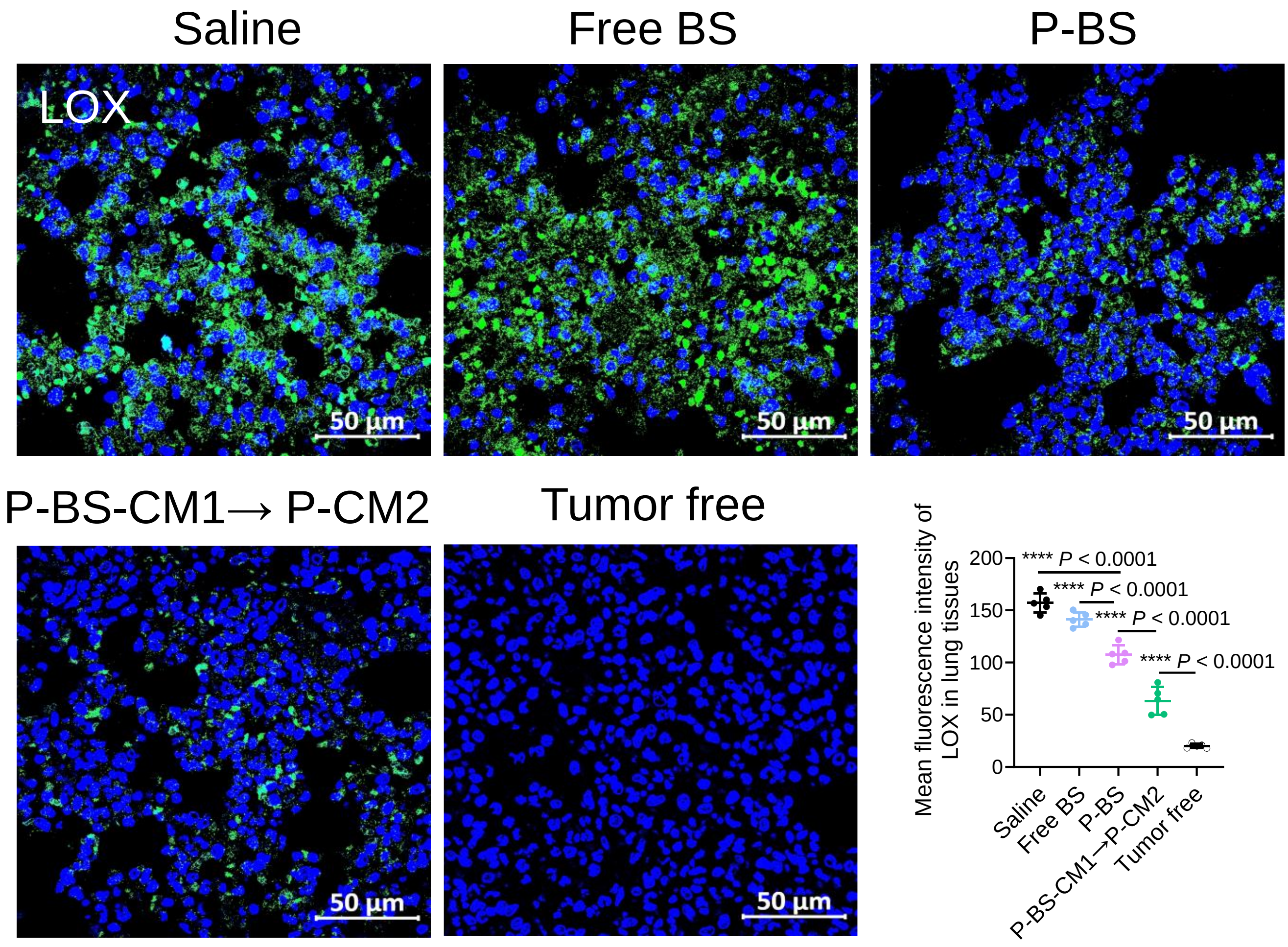

Supplementary Fig. 14 | Immunofluorescence staining and semi-quantitative analysis of typical TDSF, Lysyloxidase (LOX), in lung tissues of spontaneous lung metastasis mouse models of female BALB/c mice orthotopically bearing murine 4T1 breast tumors mice on day 28, after treatments with three cycles of free BS, P-BS, and P-BS-CM1→P-CM2 (24 h time lag) on day 7, 14, and 21. Blue, cell nuclei; green, LOX. Scale bars, 50 μm. Images are representative of five mice per group and analyzed by the Image J software. Data are presented as mean ± SD. Statistics are calculated by one-way ANOVA with Tukey's multiple comparisons test. **** $P < 0.0001$. Source data are provided as a Source Data file.

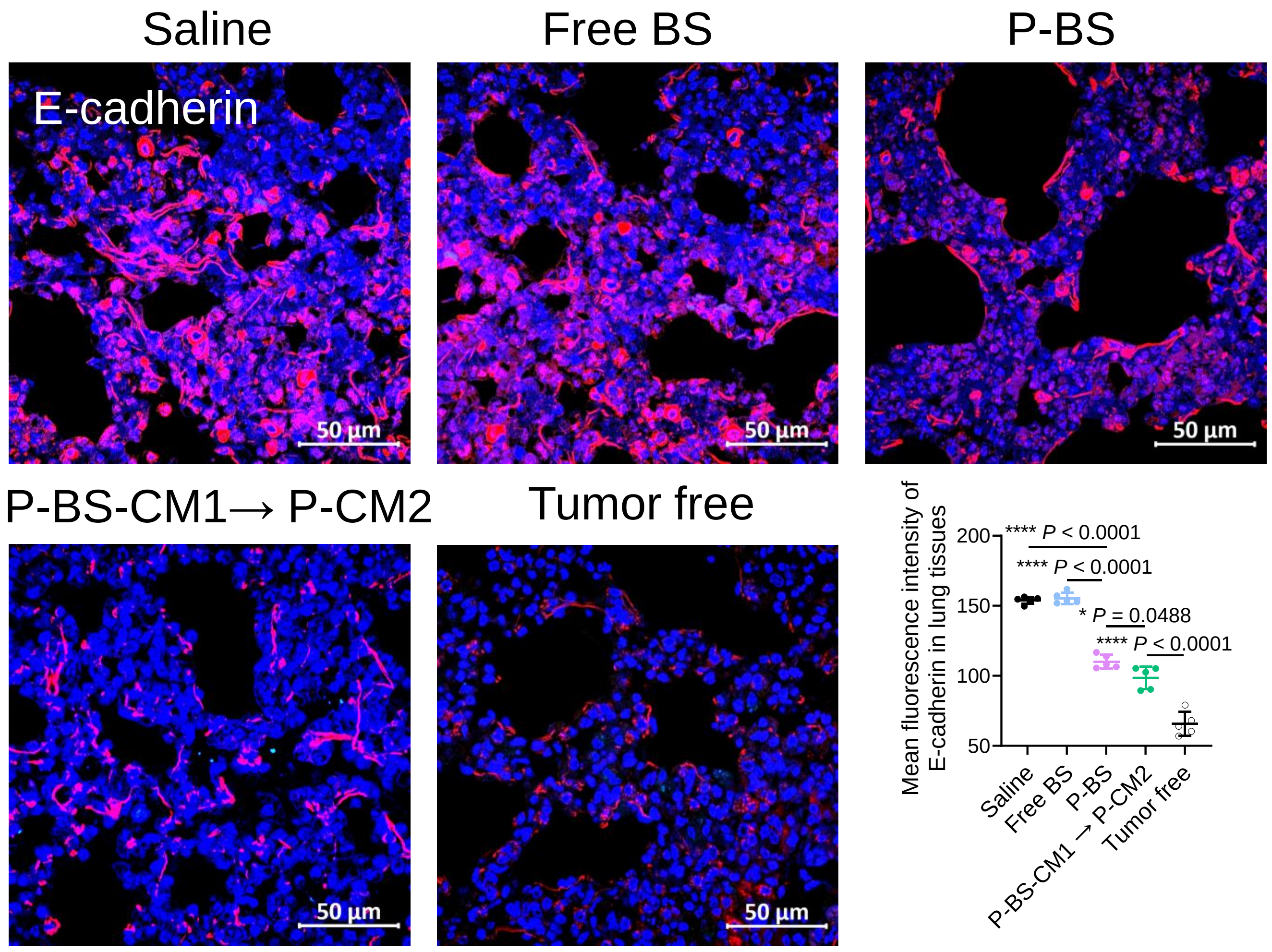

Supplementary Fig. 15 | Immunofluorescence staining and semi-quantitative analysis of E-cadherin in lung tissues of spontaneous lung metastasis mouse models of female BALB/c mice orthotopically bearing murine 4T1 breast tumors mice on day 28, after treatments with three cycles of free BS, P-BS, and P-BS-CM1→P-CM2 (24 h time lag) on day 7, 14, and 21. Blue, cell nuclei; red, E-cadherin. Images are representative of five mice per group and analyzed by the Image J software. Data are presented as mean ± SD. Statistics are calculated by one-way ANOVA with Tukey's multiple comparisons test. * $P < 0.05$, **** $P < 0.0001$. Source data are provided as a Source Data file.

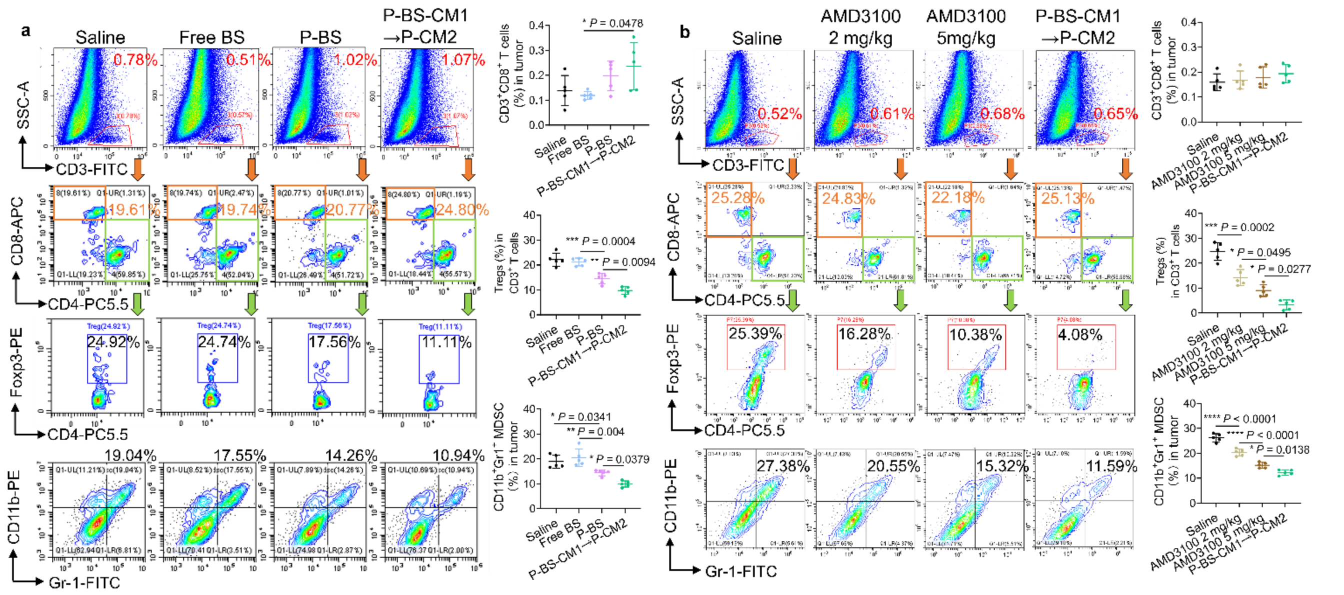

Supplementary Fig. 16 | Flow cytometry gating strategies and analyses for CD8+ T lymphocytes, regulatory T cells (Tregs) and myeloid-derived suppressor cells (MDSCs) in tumor tissues. Spontaneous lung metastasis mouse models of female BALB/c mice orthotopically bearing murine 4T1 breast tumors received **a,** three cycles of intravenously administered free BS, P-BS, or sequential P-BS-CM1→P-CM2 (24 h time lag) treatments on days 7, 14, and 21; or **b,** intraperitoneal injection of AMD3100 (2 mg/kg or 5 mg/kg) daily from day 7 to day 27. Quantification by flow cytometry and representative flow cytometry plots of $CD3^{+}CD8^{+}$ T lymphocytes, immunosuppressive Tregs ($Foxp3^{+}$, gated on $CD4^{+}$ T cells), and immunosuppressive MDSCs ($CD11b^{+}Gr1^{+}$) in tumor tissues at the endpoint on day 28. n=5 animals per group. Data are presented as mean ± SD. Statistics are calculated by one-way ANOVA with Tukey's multiple comparisons test. *$P < 0.05$, **$P < 0.01$, ***$P < 0.001$, ****$P < 0.0001$. Flow cytometry analysis is carried out with CytExpert 2.4 software. Source data are provided as a Source Data file.

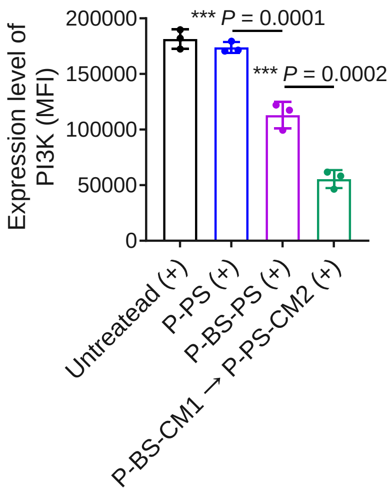

Supplementary Fig. 17 | Analysis of phosphatidylinositol 3-kinase (PI3K) pathway in 4T1 cells. 4T1 cells were either left untreated or treated with P-PS, P-BS-PS, or P-BS-CM1→P-PS-CM2, prior to flow cytometry analysis of CXCR4 downstream PI3K pathway. n=3 biologically independent samples. (+) means the presence of laser irradiation. The experiment was repeated twice independently with similar results. Data are presented as mean ± SD. Statistics are calculated by one-way ANOVA with Tukey's multiple comparisons test. *** $P < 0.001$, **** $P < 0.0001$. Source data are provided as a Source Data file.

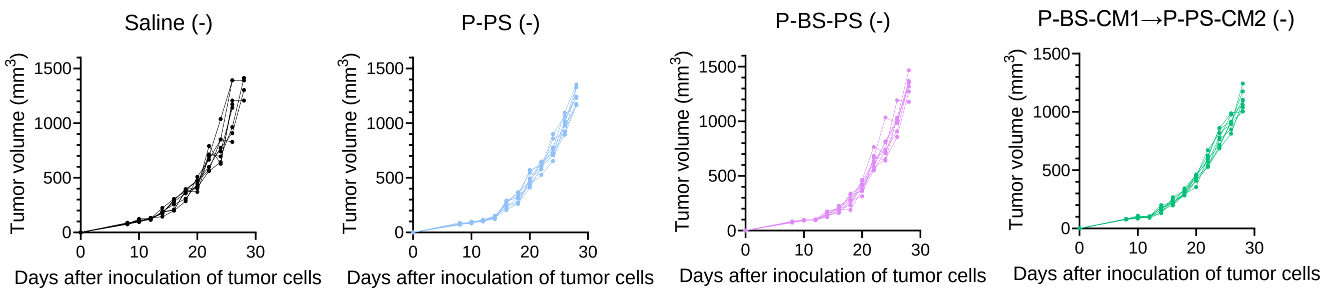

Supplementary Fig. 18 | Individual tumor growth without laser irradiation. Female BALB/c mice orthotopically bearing murine 4T1 breast tumors were treated with P-PS, P-BS-PS or P-BS-CM1→P-PS-CM2 without laser irradiation for three cycles as depicted in Fig. 8a. n=8 animals per group. Source data are provided as a Source Data file.

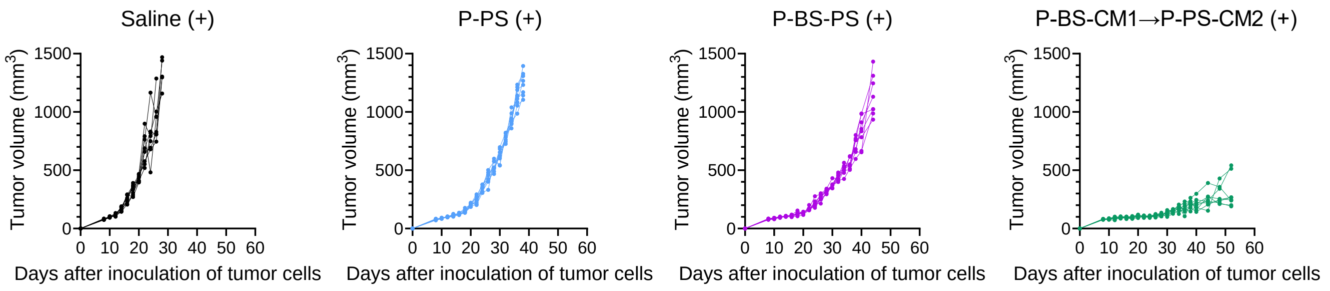

Supplementary Fig. 19 | Individual tumor growth with laser irradiation. Female BALB/c mice orthotopically bearing murine 4T1 breast tumors were treated with P-PS, P-BS-PS or P-BS-CM1→P-PS-CM2 with laser irradiation for three cycles as depicted in Fig. 8b. n=8 animals per group. Source data are provided as a Source Data file.

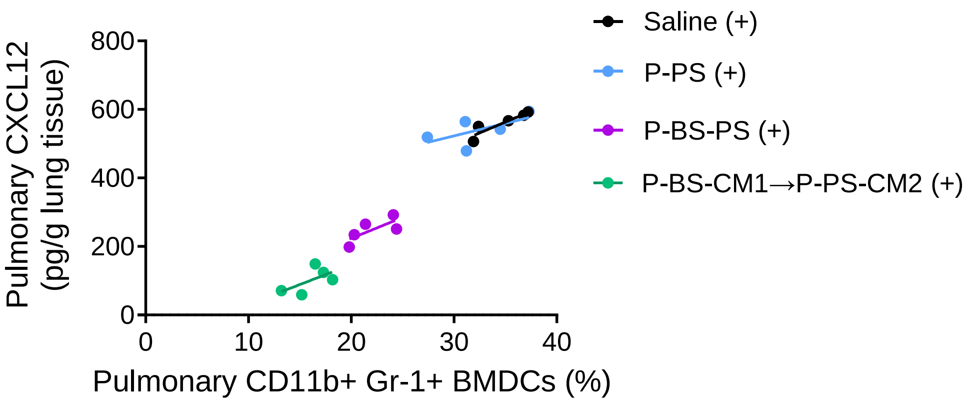

Supplementary Fig. 20 | Correlation between pulmonary bone marrow derived cells (BMDCs) and Chemokine (C-X-C Motif) ligand 12 (CXCL12). Female BALB/c mice orthotopically bearing murine 4T1 breast tumors received three weekly treatment cycles of P-PS, P-BS-PS or P-BS-CM1→P-PS-CM2 with laser irradiation starting on Day 7. n=5 animals per group. Analyses occurred on day 28. Source data are provided as a Source Data file.

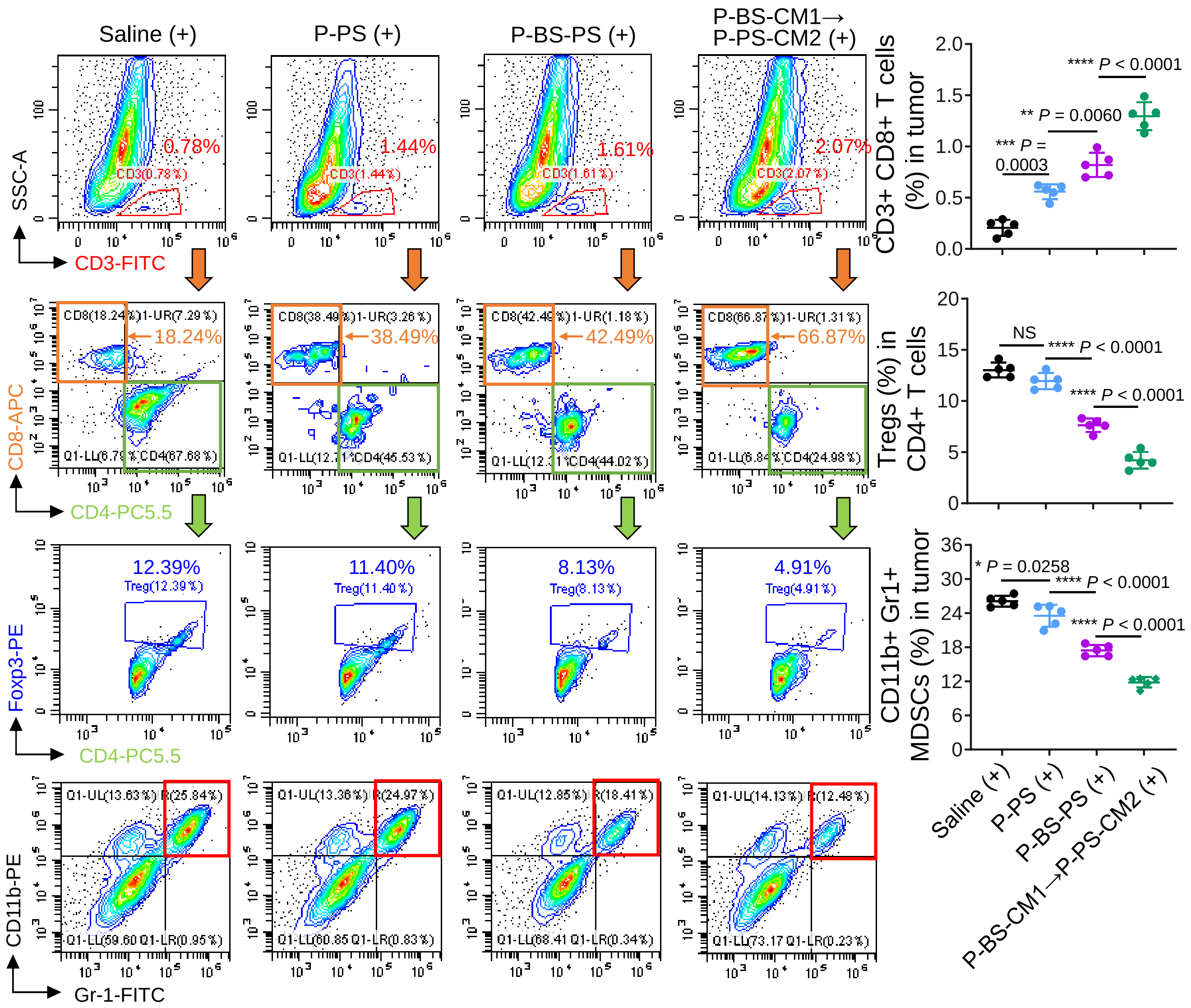

Supplementary Fig. 21 | Flow cytometry gating strategies and quantification of tumor-infiltrating CD8+ T lymphocytes, regulatory T cells (Tregs, $Foxp3^{+}CD4^{+}$), and myeloid-derived suppressor cells (MDSCs, $CD11b^{+}Gr1^{+}$) in orthotopic 4T1 tumors in female mice on Day 28 after three cycle treatments of P-PS, P-BS-PS or P-BS-CM1→P-PS-CM2 with laser irradiation. n=5 animals per group. Analyses occurred on day 28. Data are presented as mean ± SD. Statistics are calculated by one-way ANOVA with Tukey's multiple comparisons test. NS, not significant, *P < 0.05, **P < 0.01, ***P < 0.001, ****P < 0.0001. Flow cytometry analysis is carried out with CytExpert 2.4 software. Source data are provided as a Source Data file.

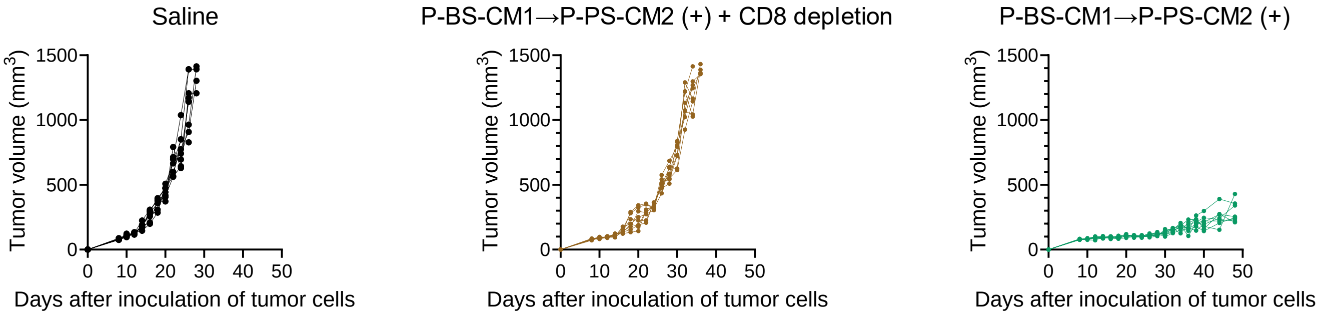

Supplementary Fig. 22 | Individual tumor growth upon $CD8^+$ T cell ablation. Female BALB/c mice orthotopically bearing murine 4T1 breast tumors were concurrently treated with P-BS-CM1→P-PS-CM2 (+) and CD8-depleting antibodies. n=8 animals per group. Source data are provided as a Source Data file.

Supplementary Table 1. Characterization of various synthetic copolymers. Data of ζ-potential and size were presented as mean ± SD. The experiments were repeated three times independently with similar results.

Conjugates	Mw (kDa)	PDI	ζ-potential (mV)	Size (nm)	BS content (wt%)	Number of BS grafts per polymer	CM content (wt%)	Number of CM grafts per polymer	Cy3, Cy5, or PS content (wt%)
P-Cy5	107.6	1.22	-3.72±1.31	8.6±1.5	-	-	-	-	0.92
P-BS	117.9	1.24	-15.53±1.45	15.7±0.8	11.1	~ 6	-	-	-
P-BS-Cy5	121.1	1.44	-17.56±0.58	14.8±1.6	11.7	~ 6	-	-	0.87
P-BS-CM1	131.1	1.39	-5.67±0.79	17.5±2.1	10.9	~ 6	12.2	~ 4	-
P-BS-CM1-Cy5	133.7	1.46	-6.27±1.21	20.1±0.7	10.8	~ 6	13.2	~ 4	0.82
P-CM2	151.2	1.21	-8.73±0.34	19.2±0.7	-	-	32.5	~ 12	-
P-CM2-Cy3	149.4	1.31	-9.21±0.67	20.4±0.5	-	-	34.8	~ 12	0.82
P-CM2-Cy5	148.8	1.27	-8.94±0.45	19.8±0.7	-	-	32.7	~ 12	0.77
P-PS	115.4	1.25	-5.02±2.19	22.2±1.7	-	-	-	-	7.12
P-BS-PS	128.9	1.45	-18.24±2.31	22.4±1.4	11.1	~ 6	-	-	6.71
P-PS-CM2	155.4	1.22	-11.21±1.24	25.8±1.2	-	-	31.4	~ 12	6.34

*Mw: molecular weight; PDI: polydispersity; BS: CXCR4 binding sequence; CM: coiled motif; PS: photosensitizer.
