# Supplementary material for: Cell surface patching via CXCR4-targeted nanothreads for cancer metastasis inhibition
Source: Nat Commun. 2024 Mar 29;15:2763. doi: 10.1038/s41467-024-47111-z (PMC10980815; doi:10.1038/s41467-024-47111-z)
Supplement: Supplementary file 3 — Reporting Summary [file 41467_2024_47111_MOESM3_ESM.pdf]

nature portfolio

Corresponding author(s): Lian Li

Last updated by author(s): Mar 9, 2024

Reporting Summary

Nature Portfolio wishes to improve the reproducibility of the work that we publish. This form provides structure for consistency and transparency in reporting. For further information on Nature Portfolio policies, see our Editorial Policies and the Editorial Policy Checklist.

Statistics

For all statistical analyses, confirm that the following items are present in the figure legend, table legend, main text, or Methods section.

n/a	Confirmed	
☐	☒	The exact sample size (*n*) for each experimental group/condition, given as a discrete number and unit of measurement
☐	☒	A statement on whether measurements were taken from distinct samples or whether the same sample was measured repeatedly
☐	☒	The statistical test(s) used AND whether they are one- or two-sided<br>*Only common tests should be described solely by name; describe more complex techniques in the Methods section.*
☒	☐	A description of all covariates tested
☒	☐	A description of any assumptions or corrections, such as tests of normality and adjustment for multiple comparisons
☐	☒	A full description of the statistical parameters including central tendency (e.g. means) or other basic estimates (e.g. regression coefficient) AND variation (e.g. standard deviation) or associated estimates of uncertainty (e.g. confidence intervals)
☐	☒	For null hypothesis testing, the test statistic (e.g. *F*, *t*, *r*) with confidence intervals, effect sizes, degrees of freedom and *P* value noted<br>*Give P values as exact values whenever suitable.*
☒	☐	For Bayesian analysis, information on the choice of priors and Markov chain Monte Carlo settings
☒	☐	For hierarchical and complex designs, identification of the appropriate level for tests and full reporting of outcomes
☒	☐	Estimates of effect sizes (e.g. Cohen's *d*, Pearson's *r*), indicating how they were calculated

Our web collection on statistics for biologists contains articles on many of the points above.

Software and code

Policy information about availability of computer code

Data collection	The size and zeta potential of copolymers were measured with Zetasizer Nano ZS90 (Malvern, England). The molecule weight of copolymers were measured with AKT purifier (GE, USA). The rheology of copolymers were measured with TA Instruments AR 2000 (USA). Scanning electron microscopy images were acquired on a electron microscopy (ThermoFisher USA). Microplate based assays were performed on Varioskan LUX microplate reader (ThermoFisher, USA). Fluorescence images were acquired usingLSM 510 DUE cofocal laser scanning microscope (Carl Zeiss, Germany) with Zen software. The images of mice and organs were collected by IVIS Spectrum imaging system (Perkin Elmer, USA). All flow data were collected by a CytoFLEX flow cytometry (Beckman Coulter, USA) with CytExpert 2.4 software.
Data analysis	Softwares used in analysis include Graphpad Prism 8, CytExpert 2.4, Image J 1.52, Microsoft Excel 2010, and DAS 2.0.

For manuscripts utilizing custom algorithms or software that are central to the research but not yet described in published literature, software must be made available to editors and reviewers. We strongly encourage code deposition in a community repository (e.g. GitHub). See the Nature Portfolio guidelines for submitting code & software for further information.

Data

Policy information about availability of data

All manuscripts must include a data availability statement. This statement should provide the following information, where applicable:

- Accession codes, unique identifiers, or web links for publicly available datasets
- A description of any restrictions on data availability
- For clinical datasets or third party data, please ensure that the statement adheres to our policy

The mass spectrometry proteomics data used in this study are avaliable in the ProteomeXchange partner repository with the dataset identifier PXD050511 [https://proteomecentral.proteomexchange.org/cgi/GetDataset?ID=PXD050511]. The remaining data are avaliable within the Article, Supplementary Information of Source Data file. Source data are provided with this paper.

Research involving human participants, their data, or biological material

Policy information about studies with human participants or human data. See also policy information about sex, gender (identity/presentation), and sexual orientation and race, ethnicity and racism.

Reporting on sex and gender	N/A
Reporting on race, ethnicity, or other socially relevant groupings	N/A
Population characteristics	N/A
Recruitment	N/A
Ethics oversight	N/A

Note that full information on the approval of the study protocol must also be provided in the manuscript.

Field-specific reporting

Please select the one below that is the best fit for your research. If you are not sure, read the appropriate sections before making your selection.

☒ Life sciences ☐ Behavioural & social sciences ☐ Ecological, evolutionary & environmental sciences

For a reference copy of the document with all sections, see nature.com/documents/nr-reporting-summary-flat.pdf

Life sciences study design

All studies must disclose on these points even when the disclosure is negative.

Sample size	Sample size (at least n = 3) was chosen on the basis of prior experiments (ACS Nano 2019, 13: 11422; Adv. Funct. Mater. 2020,30: 1908961; J. Control. Release, 2021,334: 248-262) and not predetermined by a statistical method.
Data exclusions	No data were excluded.
Replication	The experimental findings were performed at least twice independently.
Randomization	Cells and animals were randomly allocated into experimental groups.
Blinding	The investigators were blinded to group allocation during data collection and analysis.

Reporting for specific materials, systems and methods

We require information from authors about some types of materials, experimental systems and methods used in many studies. Here, indicate whether each material, system or method listed is relevant to your study. If you are not sure if a list item applies to your research, read the appropriate section before selecting a response.

Materials & experimental systems

n/a	Involved in the study
☐	☒ Antibodies
☐	☒ Eukaryotic cell lines
☒	☐ Palaeontology and archaeology
☐	☒ Animals and other organisms
☒	☐ Clinical data
☒	☐ Dual use research of concern
☒	☐ Plants

Methods

n/a	Involved in the study
☒	☐ ChIP-seq
☐	☒ Flow cytometry
☒	☐ MRI-based neuroimaging

Antibodies

Antibodies used	Alexa Fluor 647 conjugated secondary antibody (Abcam, ab150083, 1:1000), Alexa Fluor 488 conjugated secondary antibody (Abcam, ab, 1:1000), Anti-CD8a-APC (Biolegend, 100711, clone:53-6-7; 1:300), Anti-IFN-γ-PE (Biolegend, 505808, clone: XMG1.2; 1:300), Anti-CD45-PerCP/Cy5.5 (Biolegend, 103132, clone: 30-F11; 1:300), Anti-CD44-PE (Biolegend, 163610, clone: QA19A43; 1:300), Anti-CD62L-Percp/Cy5.5 (Biolegend, 161210, clone: W18021D; 1:300), Anti-CXCR2-PE (Biolegend, 149304, clone: SA044G4; 1:300), Anti-CXCR5-PE (Biolegend, 145504, clone: L138D7; 1:300), Anti-CXCR7-PE (Biolegend, 331104, clone: 8F11-M16; 1:300), Anti-Foxp3-PE (BD, 560408, clone: MF231:300), Anti-Vimentin (HUABIO, ET1610-39 clone: SC60-05, EM0401, clone: D4-B11; 1:500), Anti-PI3K (HUABIO, ET1608-70, clone: SU04-07; 1:500), Anti-TGFβ1 (HUABIO, ha721143, clone: PD00-17; 1:500), Anti-fibronectin (HUABIO, RT1224, clone: 3G4; 1:500), Anti-α-SMA (HUABIO, ET1607-53, clone: SY02-64; 1:500), Anti-calreticulin (HUABIO, ET1608-60, clone: SU37-03; 1:500), Anti-LOX (HUABIO, ET1706-31, clone: JU30-23; 1:500), Anti-HIF1α (HUABIO, ER1802-41, HA721143, clone: JE75-33; 1:500), Anti-S100A8 (Bioss, bs-2696R, 1:500), Anti-MMP9 (Bioss, bs-0397R, 1:500), Anti-CD3-FITC (Elabscience, E-AB-F1013C, clone: 17A2; 1:300), Anti-CD4-Percp/Cy5.5 (Elabscience, E-AB-F1097J, clone: GK1.5; 1:300), Anti-CD11b-PE (Elabscience, E-AB-F1081D, clone: M1/70; 1:300), Anti-Gr1-FITC (Elabscience, E-AB-F1120C, clone: RB6-8C5; 1:300), Anti-CXCR4-PE (Elabscience, E-AB-F1157D, clone:12G5; 1:300), Anti-CCR4-PE (Elabscience, E-AB-F1366D, clone: L291H4; 1:300), Anti-E-cadherin-CoraLite Plus 647 (Proteintech, CL647-65241, clone: DECMA-1; 1:300)
Validation	All antibodies were commercially obtained and have been verified by the manufacturers. Verfication data can be found by searching the manufacturer's websites using the provided catalog numbers.

Eukaryotic cell lines

Policy information about cell lines and Sex and Gender in Research

Cell line source(s)	4T1 cells (CL-0007) was provided by Procella Life Science&Technology Co., Ltd. 4T1-luc cells (YC-B004-Luc-P) was provided by Guangzhou Ubigene Biosciences Co., Ltd.
Authentication	No additional authentication was performed.
Mycoplasma contamination	All cell lines were tested for mycoplasma contamination. No mycoplasma contamination was found.
Commonly misidentified lines (See ICLAC register)	No commonly misidentified cell lines were used.

Animals and other research organisms

Policy information about studies involving animals; ARRIVE guidelines recommended for reporting animal research, and Sex and Gender in Research

Laboratory animals	Female Balb/c mice (6-8 week, 18-22 g, SPF) were provided by SPF(Beijing) Biotechnology Co., Ltd. Female mice were chosen bacause metastatic breast cancers are overwhelmingly seen in female patients. All mice were housed in a specific pathogen-free environment at 21±1 centigrade ℃ and 60±5% humidity, with a 12 hours light-dark cycle.
Wild animals	The study did not involve wild animals.
Reporting on sex	Only female mice were used.
Field-collected samples	The study did not involve samples collected from the field.
Ethics oversight	All animal experiments were approved by the Institutional Animal Care and Ethics Committee of Sichuan University, All animal experiments were conducted in the Animal Laboratory of West China School of Pharmacy in Sichuan University (accreditation number: SYXK(Chuan)2018-113). Female mice were chosen because metastatic breast cancers are overwhelmingly seen in female patients. According to the guidelines of ethics committee, the maximal tumor size permitted was 1500 mm3. Mice were euthanized when the tumor burden exceeeded this threshold.

Note that full information on the approval of the study protocol must also be provided in the manuscript.

Plants

Seed stocks	N/A
Novel plant genotypes	N/A
Authentication	N/A

Flow Cytometry

Plots

Confirm that:

- [x] The axis labels state the marker and fluorochrome used (e.g. CD4-FITC).
- [x] The axis scales are clearly visible. Include numbers along axes only for bottom left plot of group (a 'group' is an analysis of identical markers).
- [x] All plots are contour plots with outliers or pseudocolor plots.
- [x] A numerical value for number of cells or percentage (with statistics) is provided.

Methodology

Sample preparation	Mice in experimental groups were sacrificed, and organs were collected, cut into small pieces, and dissociated in digestion buffer. Then single cell suspension from tissues was obtained by filtering the buffer through a 70 µm nylon strainer, lysing red blood cells by incubating with ACK lysing buffer, washing cells twice with cold PBS, and suspending cells in PBS.
Instrument	Beckman Coulter, CytoFLEX flow cytometer
Software	CytExpert, version 2.4.0.28
Cell population abundance	At least 10,000 total events were collected for analysis.
Gating strategy	CD4+ T cells and CD8+ T cells were gated from CD3+ cells. Tregs were gated from CD3+CD4+ T cells. IFN-γ+ cells were gated from CD8+ T cells. CD44+CD62L- memory effector T cells were gated from CD8+ cells.

- [x] Tick this box to confirm that a figure exemplifying the gating strategy is provided in the Supplementary Information.
